# Based on the Metabolomic Approach the Energy Metabolism Responses of Oriental River Prawn *Macrobrachium nipponense* Hepatopancreas to Acute Hypoxia and Reoxygenation

**DOI:** 10.3389/fphys.2018.00076

**Published:** 2018-04-09

**Authors:** Shengming Sun, Zhongbao Guo, Hongtuo Fu, Xianping Ge, Jian Zhu, Zhimin Gu

**Affiliations:** ^1^Key Laboratory of Genetic Breeding and Aquaculture Biology of Freshwater Fishes, Ministry of Agriculture, Freshwater Fisheries Research Center, Chinese Academy of Fishery Sciences, Wuxi, China; ^2^Guangxi Academy of Fishery Sciences, Nanning, China; ^3^Agriculture Ministry Key Laboratory of Healthy Freshwater Aquaculture, Zhejiang Institute of Freshwater Fisheries, Huzhou, China

**Keywords:** *Macrobrachium nipponense*, metabolomics, hypoxia, hepatopancreas, oxidative stress

## Abstract

Hypoxia represents a major physiological challenge for prawns and is a problem in aquaculture. Therefore, an understanding of the metabolic response mechanism of economically important prawn species to hypoxia and re-oxygenation is essential. However, little is known about the intrinsic mechanisms by which the oriental river prawn *Macrobrachium nipponense* copes with hypoxia at the metabolic level. In this study, we conducted gas chromatography-mass spectrometry-based metabolomics studies and assays of energy metabolism-related parameters to investigate the metabolic mechanisms in the hepatopancreas of *M. nipponense* in response to 2.0 O_2_/L hypoxia for 6 and 24 h, and reoxygenation for 6 h following hypoxia for 24 h. Prawns under hypoxic stress displayed higher glycolysis-related enzyme activities and lower mRNA expression levels of aerobic respiratory enzymes than those in the normoxic control group, and those parameters returned to control levels in the reoxygenated group. Our results showed that hypoxia induced significant metabolomic alterations in the prawn hepatopancreas within 24 h. The main metabolic alterations were depletion of amino acids and 2-hydroxybutanoic acid and accumulation of lactate. Further, the findings indicated that hypoxia disturbed energy metabolism and induced antioxidant defense regulation in prawns. Surprisingly, recovery from hypoxia (i.e., reoxygenation) significantly affected 25 metabolites. Some amino acids (valine, leucine, isoleucine, lysine, glutamate, and methionine) were markedly decreased compared to the control group, suggesting that increased degradation of amino acids occurred to provide energy in prawns at reoxygenation conditions. This study describes the acute metabolomic alterations that occur in prawns in response to hypoxia and demonstrates the potential of the altered metabolites as biomarkers of hypoxia.

## Introduction

The level of dissolved oxygen is a key indicator of water quality, and partially determines the intensity of crustacean aquaculture. Much research has focused on the negative effects of hypoxia on crustaceans. At hypoxic conditions, tissues must increase anaerobic energy production, improve energy use, or lower energy consumption. The responses of crustaceans to hypoxia may result in physiological, cellular, molecular and behavioral changes that depend on the duration and level of the hypoxic stress: these changes can be seen in the behavioral responses of some species (Wu, 2002; Bell and Eggleston, 2005; Craig et al., 2005), oxygen transport (Mangum and Rainer, 1988; Mangum, 1997), growth and reproduction (Ocampo et al., 2000; Brown-Peterson et al., 2008, 2011), immune response (Qiu et al., 2011; Kniffin et al., 2014), transcriptomic responses (Li and Brouwer, 2009, 2013; Sun et al., 2015), and proteomic responses (Jiang et al., 2009; Sun et al., 2016b). However, in physiology research on aquatic invertebrates, no study to date has reported a comparative analysis of the metabolome profiles in crustaceans under hypoxic conditions.

Recently, metabolomics studies have been widely applied in aquatic animals to elucidate the biological effects of hypoxic stressors on organisms (Hines et al., 2007; Hallman et al., 2008; Tuffnail et al., 2009; Lardon et al., 2013a,b). Several analytical techniques have been well established and are frequently applied in metabolomics studies (Luo et al., 2007; Nudi et al., 2008; Shao et al., 2015; Li et al., 2017), such as liquid chromatography–mass spectrometry (LC–MS), nuclear magnetic resonance (NMR), high-performance liquid chromatography (HPLC), and gas chromatography–mass spectrometry (GC–MS). Among these analytical techniques, GC-MS is frequently used in metabolomics studies and is highly sensitive and reproducible (Ruan et al., 2013; Ren et al., 2015). Importantly, GC-MS-based metabolomics analysis has been used to investigate the biological effects of sulfide pollution in shrimp (Li et al., 2017). Their work confirmed the applicability of GC-MS-based metabolomics analysis to characterize the biological effects of environmental stressors in crustaceans.

*Macrobrachium nipponense* (Crustacea; Decapoda; Palaemonidae), also called the oriental river prawn, is an important aquaculture species that is distributed widely in freshwater and low-salinity estuarine regions in Asia (Ma et al., 2011). Prawns are relatively susceptible to hypoxia compared with most crustaceans (Sun et al., 2015). Thus, in prawn production, hypoxia may cause large economic losses because of increased mortality and decreased growth rate. In crustaceans, the functions of the hepatopancreas include carbohydrate and lipid metabolism, oxidative stress, energy storage and breakdown (Wang et al., 2008). Therefore, we hypothesized that the *M. nipponense* hepatopancreas undergoes marked metabolomic changes in response to hypoxia (Liu et al., 2014; Song Q. et al., 2017). Nonetheless, the specific mechanisms by hepatopancreas of prawns respond to hypoxia stress are largely unknown and hypoxia-related metabolomics information remains limited. Thus, the effects of hypoxia and subsequent recovery on *M. nipponense* hepatopancreas were investigated using a GC-MS-based metabolomics approach. We also compared the activities of metabolic enzymes and electron transport chain-related gene expression level changes induced by hypoxia between *M. nipponense* and other species. This study provides insight into the metabolic pathways of *M. nipponense* that are affected by acute hypoxia and reoxygenation.

## Materials and methods

### Experimental prawn

All experimental procedures involving prawn were approved by the institution animal care and use committee of the Chinese Academy of Fishery Sciences. Healthy *M. nipponense* (wet weight 2.12–3.86 g) were obtained from Dapu experimental base near by Tai Lake, the Freshwater Fisheries Research Center of the Chinese Academy of Fishery Sciences (Wuxi, China). The prawns were acclimated in 12,300-L aerated freshwater tanks for 1 week and fed commercial flake food twice per day. The culture conditions were: 23.5 ± 0.5°C, pH 8.3 ± 0.09, 6.8 ± 0.2 mg/L dissolved oxygen, and < 0.1 mg/L total ammonia-nitrogen. The prawn were raised under the natural photoperiod.

### Hypoxia and recovery stress

The control group was maintained in normoxic conditions (6.5 ± 0.2 mg O_2_/L). In the hypoxia groups, severe hypoxic conditions (2.0 ± 0.1 mg O_2_/L) for 24 h within the treatment tanks were maintained by adding N_2_ gas until the desired O_2_ concentrations were reached (Sun et al., 2014); oxygen levels were maintained by adding N_2_ gas when needed. The hypoxic DO value was chosen based on previous observations of juvenile oriental river prawn. DO and temperature were measured using a water-quality instrument (YSI Inc., Yellow Springs, OH, USA). Six hundred prawns were randomly allocated to 12 tanks (four treatments were conducted in triplicate): hypoxia for 0 h (control), severe hypoxia for 6 h, severe hypoxia for 24 h, or severe hypoxia for 24 h followed by recovery in normoxic conditions for 6 h. The mortality rate was 10% during experiment period. The hepatopancreas samples in each group (in triplicate) were also collected for the biochemical and gene expression assays (*n* = 9), and the other hepatopancreas samples in each group (in triplicate) were also stored at −80°C until the metabolomics assays were conducted (*n* = 8). All animal experiments were conducted in accordance with the Guidelines for Experimental Animals of the Ministry of Science and Technology (Chen et al., 2017). All experimental procedures were approved by the Animal Care and Use Committee of the Chinese Academy of Fishery Sciences.

### Hepatopancreas metabolomics analysis

Samples (0.05 g, *n* = 8) in each group were extracted with 0.4 mL methanol-chloroform (v:v, 4:1). l-2-chlorophenylalanine (20 μL) was added as an internal standard and then centrifuged (11,000 × g, 15 min, 4°C). The supernatant was then transferred to a 2-ml GC/MS glass vial, and 15 μL from each sample were analyzed for quality control purposes. The hepatopancreas extracts were processed using a method described in a recent study (Li et al., 2017).

GC-time-of-flight (TOF)-MS analysis was conducted using an Agilent 7890 GC system coupled with a Pegasus HT TOF-MS, as previously described (Li et al., 2017). Each sample in the present study was analyzed eight times. Chroma TOF4.3X software (LECO Corporation, USA) and the LECO-Fiehn Rtx5 database were used for raw peak extraction, data baseline filtering and calibration, peak alignment, deconvolution analysis, peak identification, and integration of peak areas (Kind et al., 2009). Metabolomics data have been deposited to the EMBL-EBI MetaboLights database (Haug et al., 2013) with the identifier MTBLS481. The complete dataset can be accessed https://www.ebi.ac.uk/metabolights/MTBLS584.

### Analysis of differential metabolites

Three-dimensional data including the peak number, sample name, and normalized peak area were processed using the SIMCA14 software package (Umetrics, Umea, Sweden) for principal component analysis (PCA) and orthogonal projections to latent structures-discriminate analysis (OPLS-DA). PCA showed the distribution of the original data. OPLS-DA showed a higher level of group separation and thus resulted in a better understanding of the variables responsible for classification (i.e., the differences between groups). We refined this analysis by obtaining the first principal component of variable importance projection values, with values >1.0 selected as changed metabolites. In step 2, the remaining variables were then assessed using Student's *t*-test (*P* < 0.05) (Storey and Tibshirani, 2003; Ren et al., 2015; Song T. et al., 2017). Metabolites and their biological roles and pathways were identified using databases including Chemical Entities of Biological Interest (http://www.ebi.ac.uk/chebi/init.do), the Kyoto Encyclopedia of Genes and Genomes (KEGG) (http://www.genome.jp/kegg/), and National Institute of Standards and Technology (NIST) (http://www.nist.gov/index.html). KEGG pathway analysis of different metabolites was performed using Metabo Analyst 3.0 software (http://www.metaboanalyst.ca/MetaboAnalyst/).

### RNA extraction and quantitation of gene expression

Total RNA was extracted from hepatopancreas (~100 mg) using 1 mL of Trizol reagent following the manufacturer's protocol (TaKaRa, Japan). cDNAs were synthesized from 1 μg total DNA-free RNA using the PrimeScript RT reagent kit (TaKaRa, Japan). Quantitative real time-PCR was performed on a Bio-Rad iCycler iQ5 Real-Time PCR system, and β-actin was used as a reference gene (Sun et al., 2016a). Table 1 shows the primers used. The reaction was amplified with 35 cycles of 94°C for 30 s, 50°C for 30 s, and 72°C for 1 min, which were followed by 10 min of incubation at 72°C as a final extension step (Qiao et al., 2015). Dissociation curve analysis of the amplification products was performed at the end of each PCR reaction. mRNA expression levels were determined using the 2^−ΔΔCT^ method (Livak and Schmittgen, 2001).

**Table 1:** Primers used for qPCR.

**Target mRNA**	**Sequence (5′-3′)**
Cytochrome *c* oxidase subunit I-F	TATTAGGAGCGCCAGACATAGC
Cytochrome *c* oxidase subunit I-R	GGGGTAGACAGTTCATCCTGTG
ATPase subunit α-F	AGGTATCCTTGGCCGTGTTG
ATPase subunit α-R	TTGCCAGTCTGACGATCACC
ATPase subunit β-F	TGAGGTCAACTTTCCCCGAC
ATPase subunit β-R	CCTGGGCCAAACTTCTTGAC
β-actin-F	AATGTGTGACGACGAAGTAG
β-actin-R	GCCTCATCACCGACATAA

### Analysis of enzyme activity

Hepatopancreas samples were homogenized (w:v, 1:10) in ice-chilled 0.86% saline buffer at 60 Hz for 30 s, and then centrifuged at 3,000 × g for 10 min at 4°C. The supernatant was collected for further analysis. The succinate dehydrogenase (SDH), hexokinase (HK), pyruvate kinase (PK), and lactate dehydrogenase (LDH) activities of each sample were determined using commercial kits (Nanjing Jiancheng Bioengineering Institute, Nanjing, China), including succinate dehydrogenase assay kit (A022, colorimetric method, 50 tubes), hexokinase assay kit (A077-1, ultraviolet colorimetric method, 30 tubes), pyruvate kinase assay kit (A076-1, ultraviolet colorimetric method, 50 tubes), and lactate dehydrogenase assay kit (A020-2, microplate method, 96 tubes).Protein concentration in the samples was determined according to the Bradford method 1976, with bovine serum albumin as the standard.

### Statistical analysis

Data are presented as the mean ± SE values (*n* = 9). Data were transformed if necessary after evaluating assumptions of normality, equality of variances and outliers, and subjected to one-way analysis of variance (ANOVA) using the software SPSS 19.0 (International Business Machines Corporation, Armonk, NY, USA) for Windows, with post-hoc comparison of means using the Turkey-Kramer HSD test.

## Results

### Hepatopancreas metabolomic profile of *M. nipponense* in response to hypoxia and reoxygenation

**Figure 1:**
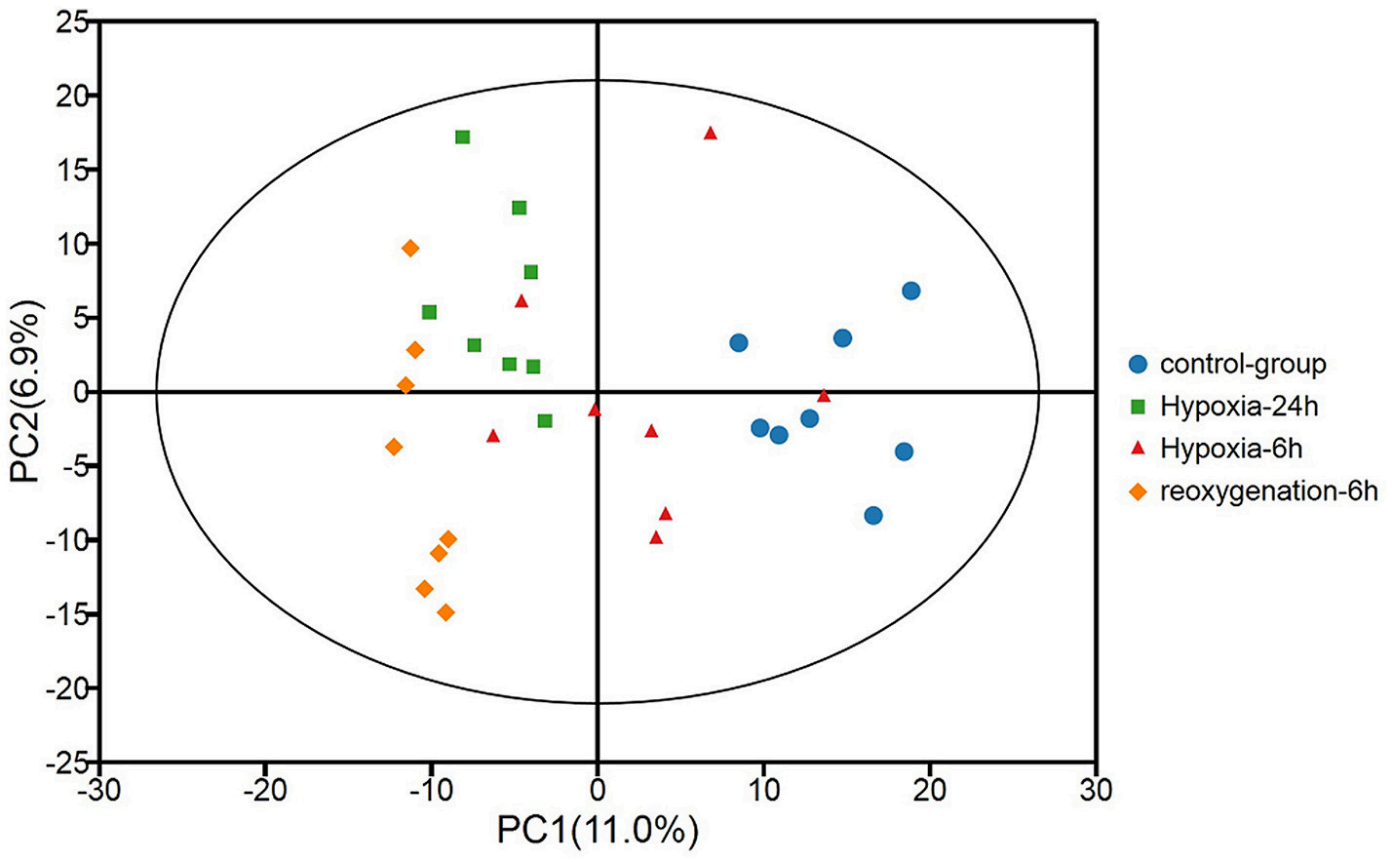
Principal component analysis (PCA) of metabolic profiles of the hepatopancreas of oriental river prawns in the control group, in response to hypoxia for 6 and 24 h, and in response to hypoxia for 24 h followed by reoxygenation for 6 h (eight biological replicates). *R*^2^X [1] = 0.111, *R*^2^X [2] = 0.179, Ellipse: Hotelling's T^2^ (95%).

**Figure 2:**
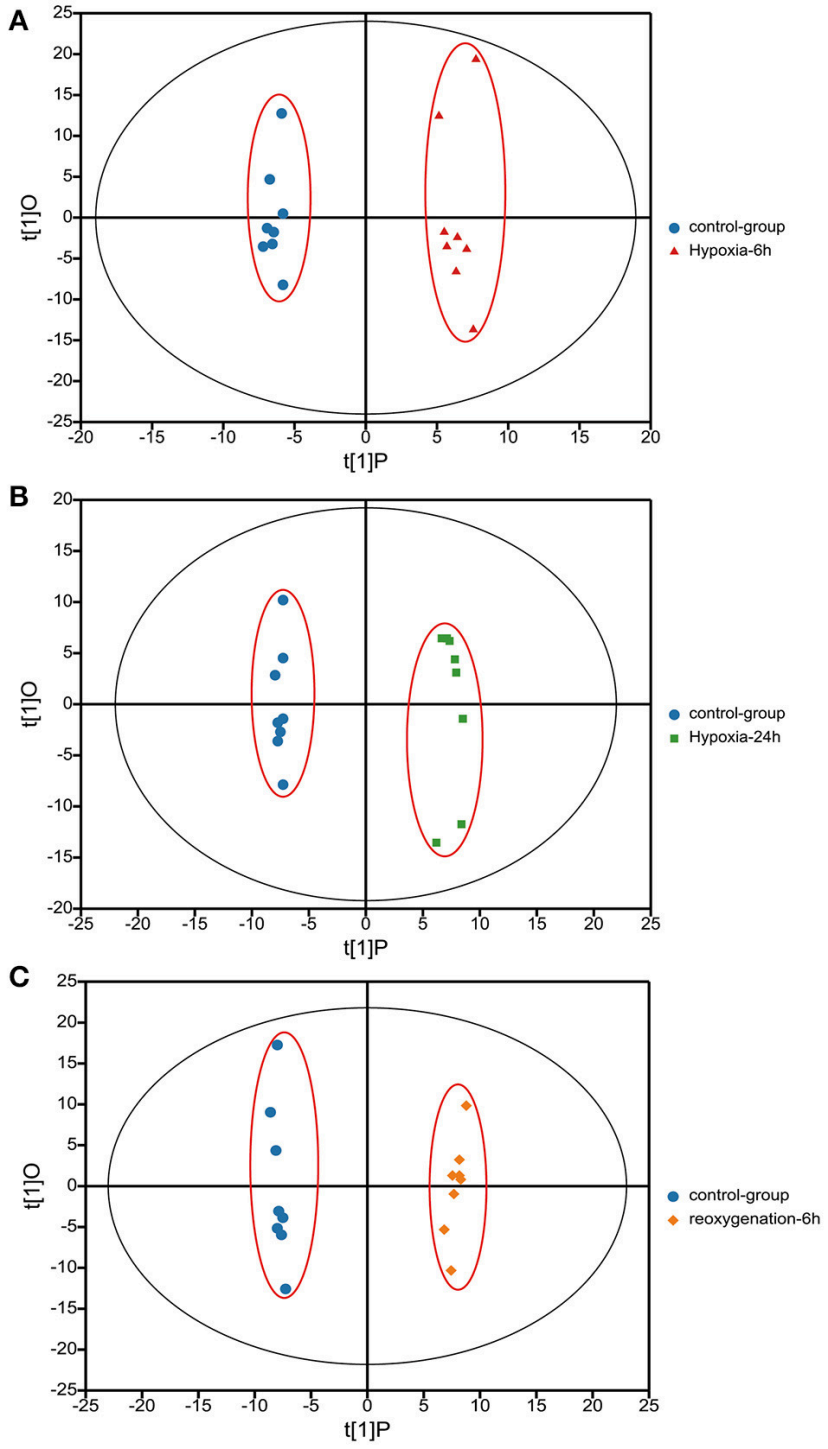
Orthogonal projections to latent structures-discriminate analysis (OPLS-DA) score plots based on the GC-MS spectra of hepatopancreas samples from *M. nipponense* in response to hypoxia for 6 h relative to the control **(A)**, hypoxia for 24 h relative to the control **(B)**, and hypoxia for 24 h followed by reoxygenation for 6 h relative to the control **(C)**. “O” means “Orthogonal” and “P” means “Predictive” in OPLS-DA. *R*^2^X [1] = 0.139, 0.127, 0.094, *R*^2^X [2] = 0.125, 0.97, 0.151. Ellipse: Hotelling's T^2^ (95%).

Metabolic profiles of prawn hepatopancreas acquired in present study were shown in Figure S1. PCA analysis of GC-TOF/MS metabolic profiles of hepatopancreas showed in Figure 1, a certain trend in metabolite shift was observed among different groups in PC1. Due to the relatively short period of hypoxia stress compared to the lifespan of a river prawn, the major metabolite pathways might remain generally stable. As a result, we believe that the metabolic shift only covered a small portion of the total metabolome data in this experiment. OPLS-DA was also applied to each sample (hypoxia or reoxygenation), and data were compared with the control group (Figure 2). The classification parameters for the software were as follows: *R*^2^Y = 0.988 and *Q*^2^Y = 0.502, *R*^2^Y = 0.994 and *Q*^2^Y = 0.698, and *R*^2^Y = 0.996 and *Q*^2^Y = 0.777 for 6-h hypoxia, 24-h hypoxia and 6-h reoxygenation, respectively. Seven-fold cross-validation was used to estimate the robustness and predictive ability of our model, and permutation tests were used to further validate the model. The *R*^2^ and *Q*^2^ intercept values were 0.97, 0.95, 0.94, and −0.34, −0.55, −061, respectively, after 200 permutations for different treatments. The low *Q*^2^ intercept values indicate the robustness of the models, the low risk of overfitting and the reliability of the method. The OPLS-DA score plots of the first and second principal components (t [1] P and t [1] O) showed that the prawns under hypoxia for 6 h, hypoxia for 24 h, and hypoxia for 24 h followed by reoxygenation for 6 h were clearly separated from those in control conditions in the direction of t [1] P. Thus, the spectral characteristics of the three experimental groups were markedly different from those of the control group.

Table 2 demonstrates that in the hepatopancreas of prawns, as a consequence of 6-h hypoxia, only 13 metabolites were altered (10 were upregulated and three were downregulated). In response to hypoxia for 24 h, the level of 20 metabolites changed significantly in relation to the control (seven were upregulated and 13 were downregulated) (Table 3). The main biochemicals were free amino acids and factors associated with the citrate cycle, glycolysis and redox homeostasis. In response to reoxygenation for 6 h following hypoxia for 24 h, the level of 25 metabolites changed significantly in relation to the control samples (20 were upregulated and five were downregulated) (Table 4). We observed significant changes in energy substrates and cofactors and the biochemicals associated with amino acid metabolism and glutathione synthesis.

**Table 2:** Significantly changed metabolites in *M. nipponense* hepatopancreas between the control group and the 6-h hypoxia group.

**Peak**	**VIP^a^**	***P*-value^b^**	**FC^c^**	**LOG FC^d^**	**Metabolic pathway**
Valine	2.67	3.2778E-05	3.49	1.80	Amino metabolism
Adenine	1.97	0.02	0.68	−0.55	Amino metabolism
Lactic acid	2.51	3.3799E-05	2.29	1.20	Energy metabolism
Choline	1.03	0.016	0.34	−1.54	Amino metabolism
Gamma-aminobutyric acid	2.14	0.006	2.12	1.09	Amino metabolism
Taurine	2.26	0.005	2.16	1.11	Amino metabolism
Succinic acid	2.46	0.043	0.00002	−15.672	Energy metabolism
2-hydroxybutanoic acid	2.46	0.000	2.63	1.40	Glutathione synthesis
Isoleucine	2.07	0.014	1.26	0.33	Energy metabolism
Citric acid	2.34	0.000	2.06	1.04	Energy metabolism
Malic acid	1.79	0.030	1.44	0.52	Energy metabolism
Alanine	1.75	0.034	4.15	2.05	Amino metabolism
Leucine	1.86	0.026	1.65	0.73	Amino metabolism

a *Variable importance in the projection (VIP) was acquired from the OPLS-DA model with a threshold of 1.0*.

b *P-values were calculated from two-tailed Student's t-tests*.

c *FC: fold change between 6-h hypoxia treatment and the control group*.

d *Positive values indicate higher levels in the 6-h hypoxia group than in the control group, negative values indicate lower levels in the 6-h hypoxia group than in the control group*.

**Table 3:** Significantly changed metabolites in *M. nipponense* hepatopancreas between the control group and the 24-h hypoxia group.

**Peak**	**VIP^a^**	***P*-value^b^**	**FC^c^**	**LOG FC^d^**	**Metabolic pathways**
Valine	1.44	0.01	0.26	−1.94	Amino metabolism
Citrulline 1	1.14	0.04	1.73	0.79	Amino metabolism
Cholesterol	2.53	8.2246E-09	4.49	2.17	Lipid metabolism
Uric acid	1.78	0.01	1.73	0.79	Purine metabolism
Succinic acid	2.14	0.04	2.0812E-05	−15.55	Energy metabolism
Citric acid	1.85	0.02	2.2245E-05	−15.46	Energy metabolism
Lysine	2.80	2.6343E-06	3.1941E-06	−18.26	Amino metabolism
Linoleic acid	1.52	0.03	0.63	−0.67	Lipid metabolism
Taurine	2.44	0.00	2.2863E-05	−15.42	Amino metabolism
Choline	1.23	0.04	0.16	−2.43	Amino metabolism
Pyruvic acid	1.38	0.01	4.11	2.04	Energy metabolism
Fumaric acid	2.21	2.3034E-05	2.73	1.45	Energy metabolism
Malic acid	1.15	0.03	0.16	−2.68	Energy metabolism
Isoleucine	1.85	0.02	0.39	−1.35	Amino metabolism
Lactose	1.18	0.05	4.24	2.08	Energy metabolism
Alanine	1.04	0.04	0.29	−1.76	Amino metabolism
Leucine	1.22	0.01	0.45	−1.15	Amino metabolism
Lactic acid	2.47	0.00	11833.791	13.53	Energy metabolism
Adenine	1.45	0.00	0.23	−2.13	Amino metabolism
2-hydroxybutanoic acid	1.37	0.04	0.40	−1.33	Glutathione synthesis

a *Variable importance in the projection (VIP) was acquired from the OPLS-DA model with a threshold of 1.0*.

b *P-values were calculated from two-tailed Student's t-tests*.

c *FC: fold change between 24-h hypoxia treatment and the control group*.

d *Positive values indicate higher levels in the 24-h hypoxia group than in the control group, negative values indicate lower levels in the 24-h hypoxia group than in the control group*.

**Table 4:** Significantly changed metabolites in *M. nipponense* hepatopancreas between the control group and the 24-h hypoxia followed by 6-h reoxygenation group.

**Peak**	**VIP^a^**	***P*-value^b^**	**FC^c^**	**LOG FC^d^**	**Metabolic pathways**
Alanine	2.20	1.6326E-05	2.97	1.57	Amino metabolism
Sarcosine	1.76	0.01	2.39	1.26	Energy metabolism
Galactosamine	2.05	0.03	3.14	1.65	Glycometabolism
Lactic acid	2.16	6.0506E-05	2.57	1.36	Energy metabolism
Glycine 2	1.51	0.03	0.76	−0.40	Amino metabolism
Glutamate	2.14	0.00	2.08	1.05	Amino metabolism
Succinate acid	1.56	0.02	1.34	0.42	Energy metabolism
Citric acid	1.67	0.01	1.56	0.64	Energy metabolism
Lysine	1.20	0.02	0.58	−0.78	Amino metabolism
Linoleic acid	1.79	0.02	2.46	1.30	Lipid metabolism
Ribose	1.42	0.02	3.17	1.66	Nucleic metabolism
Methionine	1.80	0.02	0.07	−3.83	Amino metabolism
Allose	2.30	1.2542E-05	1.64	0.72	Glycometabolism
N-ethylmaleamic acid	2.24	4.8838E-05	1.64	0.71	Glutathione synthesis
Allo-inositol	2.10	0.01	2906.92	11.51	Glycometabolism
Isoleucine	1.28	0.01	0.42	−1.26	Amino metabolism
Leucine	1.48	0.025	0.37	−1.42	Amino metabolism
Glucose	2.67	0.00	29830.06	14.86	Energy metabolism
Lactone	1.49	0.04	574.16	9.17	Energy metabolism
Pyroglutamic acid	1.47	0.02	3.40	1.76	Glutathione synthesis
Fumaric acid	1.45	0.00	3.90	1.96	Energy metabolism
Malic acid	1.56	0.03	2.29	1.19	Energy metabolism
Cysteine	1.60	0.01	1.43	0.52	Energy metabolism
Myo-inositol	1.73	0.02	2426.09	11.24	Glycometabolism
2-hydroxybutanoic acid	1.55	0.02	1.46	0.55	Glutathione synthesis

a *Variable importance in the projection (VIP) was acquired from the OPLS-DA model with a threshold of 1.0*.

b *P-values were calculated from two-tailed Student's t-tests*.

c *FC: fold change between 6-h reoxygenation treatment group and the control group*.

d *Positive values indicate higher levels in the 6-h reoxygenation treatment group than in the control group, negative values indicate lower levels in the 6-h reoxygenation treatment group than in the control group*.

### KEGG pathway analysis

**Figure 3:**
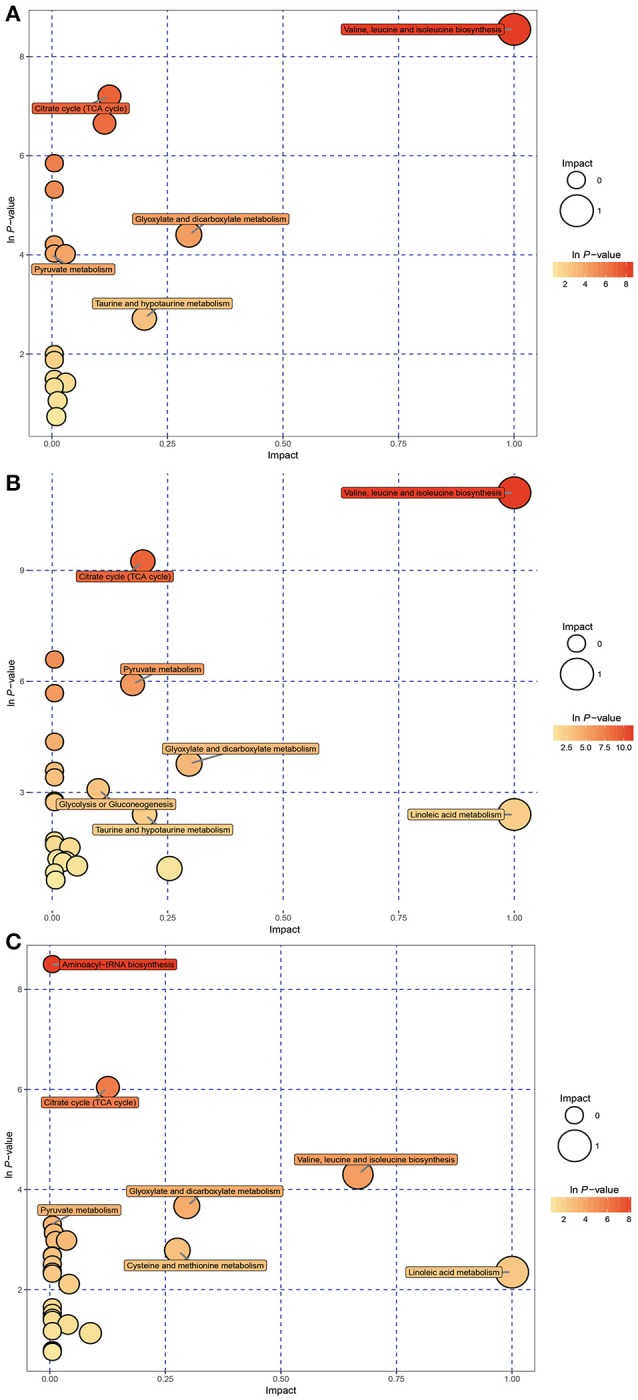
Metabolome view map of significant metabolic pathways characterized in hepatopancreas of prawns in response to hypoxia for 6 h **(A)**, hypoxia for 24 h **(B)**, and hypoxia for 24 h followed by reoxygenation for 6 h **(C)**. This figure illustrates significantly changed pathways based on enrichment and topology analysis. Larger sizes and darker colors represent greater pathway enrichment and higher pathway impact values, respectively.

KEGG pathway analysis was performed using Metabo Analyst 3.0 and differentially affected metabolites. Functional pathway analysis revealed the most relevant pathways affected by hypoxic stress included the citrate cycle (TCA cycle), pyruvate metabolism, glycolysis or gluconeogenesis, propanoate metabolism, valine, leucine, and isoleucine biosynthesis, and purine metabolism (Figures 3A,B). The significantly changed pathways identified from affected metabolites in hepatopancreas of prawns after hypoxia for 24 h followed by reoxygenation for 6 h included the TCA cycle, pyruvate metabolism, cysteine and methionine metabolism, and linoleic acid metabolism (Figure 3C).

### mRNA expression profiles of aerobic metabolism-related genes

**Figure 4:**
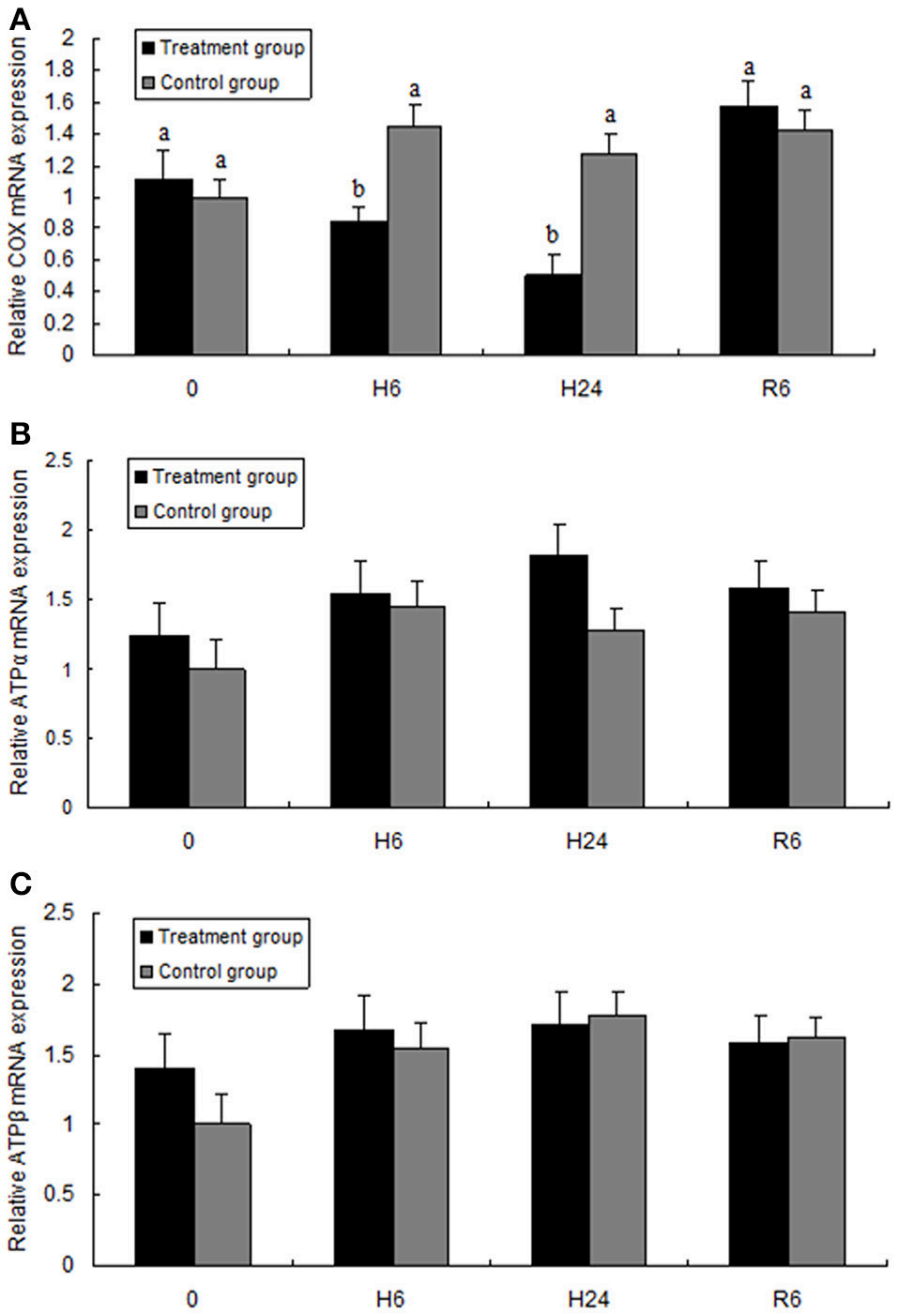
Quantitative real-time PCR analysis of **(A)** cytochrome c oxidase subunit I (COX I), **(B)** ATP synthase subunit α (ATPα), and **(C)** ATP synthase subunit β (ATPβ) mRNA expression in the hepatopancreas of juvenile oriental river prawn after exposure to hypoxia and reoxygenation. H6 = hypoxia for 6 h, H24 = hypoxia for 24 h, and R6 = hypoxia for 24 h followed by reoxygenation for 6 h. Significant differences (*P* < 0.05) among all treatment groups are indicated with different letters.

Figure 4 shows the expression levels of cytochrome *c* oxidase subunit I (COX I) and ATP synthase subunits α and β (ATPα, ATPβ). The expression level of COX I mRNA was significantly lower in the 6- or 24-h hypoxia groups than in the other groups (*F* = 8.159, *P* < 0.05). However, after hypoxia followed by reoxygenation for 6 h, the expression level increased to the control levels. The mRNA expression levels of ATPα and ATPβ had no significant change between the control (normoxia) and hypoxia groups, and no significant differences were observed in the expression levels of ATPα and ATPβ between the control (normoxia) and hypoxia–reoxygenation groups.

### Activities of energy metabolism-related enzymes of *M. nipponense* in response to hypoxia and reoxygenation

**Figure 5:**
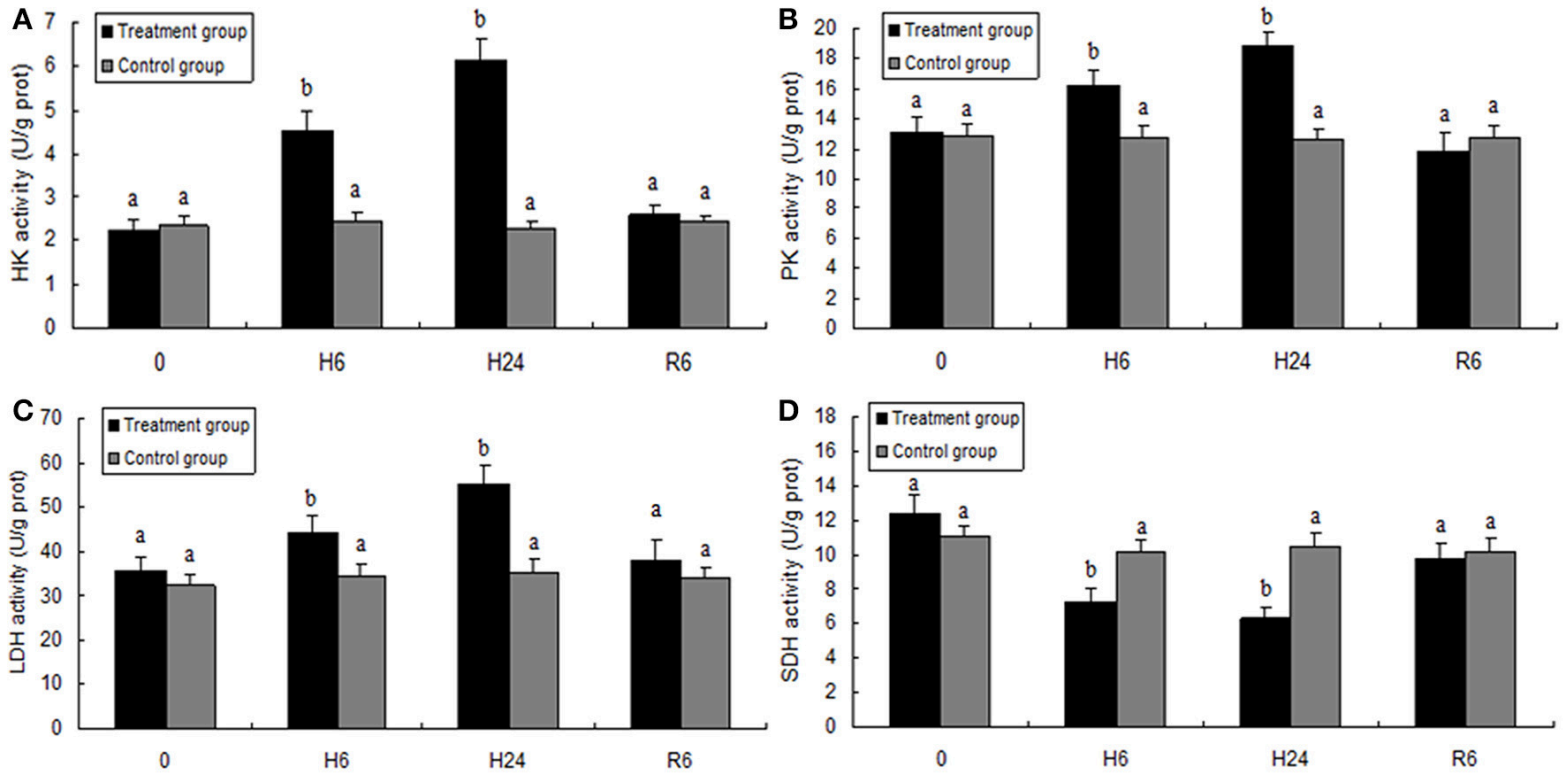
Enzyme activities of hexokinase (HK, **A**), pyruvate kinase (PK, **B**), lactate dehydrogenase (LDH, **C**), and succinate dehydrogenase (SDH, **D**) in the hepatopancreas of juvenile oriental river prawn after exposure to hypoxia and reoxygenation. H6 = hypoxia for 6 h, H24 = hypoxia for 24 h, and R6 = hypoxia for 24 h followed by reoxygenation for 6 h. Significant differences (*P* < 0.05) among all treatment groups are indicated with different letters.

HK, PK, and LDH activities were significantly higher in the hepatopancreas of *M. nipponense* in the 6- and 24-h hypoxia groups than in the other treatment groups (*F* = 600.832, *P* < 0.05; *F* = 478.105, *P* < 0.05; *F* = 741.874, *P* < 0.05, respectively), but no significant differences were observed in these parameters between the reoxygenation group and the control groups (Figure 5). SDH activity significantly decreased with increase in hypoxia time (*F* = 76.680, *P* < 0.05), but SDH activity in the reoxygenated group returned to the levels in the control group (Figure 5).

## Discussion

Crustaceans often exposed to hypoxia show a complex and highly integrated series of metabolic responses to maintain cellular homeostasis, and ATP synthesis/hydrolysis is crucial in the process of hypoxia-induced stress.

Compared with hypoxia sensitive *M. nipponense*, the survival of penaeid shrimp is not greatly affected by long-term moderate hypoxia, as reported for Pacific whiteleg shrimp *Litopenaeus vannamei* (Racotta et al., 2002), thus, we compared the mRNA expression levels of electron transport chain-related enzymes between *M. nipponense* and *L. vannamei*. First, we consider aerobic metabolism. The mitochondrial F_O_F_1_ ATP-synthase complex catalyzes ATP synthesis (Pedersen, 2007) via chemiosmotic coupling to the respiratory chain. We observed no significant changes in ATPα and ATPβ mRNA expression in prawns during hypoxia or subsequent reoxygenation; this is not consistent with previous findings in the *L. vannamei* (Martinez-Cruz et al., 2011) where mRNA level of ATPβ increased in response to hypoxia and a subsequent decreased when *L. vannamei* were re-oxygenated. A reasonable explanation is that prawns starts to use anaerobic metabolism during acute hypoxia, so it might not need to synthesize more new ATP-synthase complexes. The respiratory chain complex II (SDH) is the only enzyme of the tricarboxylic acid cycle (TCA) in the inner mitochondrial membrane, and SDH activity reflects the level of aerobic metabolism (Gao et al., 2016). Similarly with previous study in *M. nipponense* (Guan et al., 2010), the present results showed that significantly lower SDH activity was found in the *M. nipponense* exposed to hypoxia, indicating a lower level of aerobic metabolism in these animals. Inhibition of the mRNA expression of respiratory chain complex IV (COX), which we observed here in the hypoxic samples (Jimenez-Gutierrez et al., 2013, 2014), can affect the respiratory function of mitochondria, leading to cell hypoxia or death (Reiffienstcin et al., 1992). Finally, an increase in the beta-hydroxybutyric acid concentration was observed in all hypoxic groups; this probably reflects cessation of ketone-body metabolism as the TCA cycle is not functioning during hypoxia (Mimura and Furuya, 1995). Collectively, our data suggest a hypoxia-associated general inhibition of aerobic energy metabolism in *M. nipponense*.

In the present study, glycolysis-related enzyme activities of *M. nipponense* (HK, PK, and LDH) were significantly increased by hypoxia for 6 and 24 h compared with the control group, which was similar to the findings in a previous study of the white shrimp *Litopenaeus vannamei* (Soñanez-Organis et al., 2011, 2012; Cota-Ruiz et al., 2015). These findings suggest that hypoxia results in a shift from aerobic to anaerobic metabolism, and that sufficient ATP can be generated only by upregulating oxygen-independent mechanisms in hypoxic crustacea. Eventually, glucose/glycogen become exhausted and metabolic waste products such as lactate accumulate, as was also shown in our present study. Acute hypoxia caused a significant increase in the lactate concentration in the hepatopancreas extracts. Interestingly, reoxygenation treatment resulted in a higher lactate content in the hepatopancreas compared to the normoxia group; this indicates the presence of a Warburg effect-like response, as has been reported in other crustacean species (Su et al., 2014). In hypoxia, a sufficient and possibly augmented supply of glucose is required because anaerobic glycolysis is increased to obtain sufficient energy.

Levels of branched-chain amino acids including valine, isoleucine, leucine, decreased in hepatopancreas samples under 24-h hypoxia (relative to the normoxic controls), and branched-chain amino acids were still present at a lower level in prawns that were exposed to hypoxia for 24 then reoxygenated for 6 h. These essential amino acids contribute to global regulation of growth and metabolism (Wang et al., 2011). For example, a decreased concentration of isoleucine in the hepatopancreas was observed in a previous study of hypoxia in prawns and was possibly due to difficulty in taking up food because of the hypoxia (Ren et al., 2014), suggesting that hypoxia can suppress feeding behavior of prawns to some extent. Lysine is an essential amino acid for protein synthesis and a ketogenic amino acid (Sauer et al., 2015); the concentration of lysine was significantly decreased in prawns in response to hypoxia compared to the control group, suggesting that protein degradation was likely to be higher in the hypoxia group than in the control group. This could partly explain the slower growth trend of shrimp under hypoxic stress (Duan et al., 2013). The protective effect of taurine during acute hypoxia in mammalian tissues has recently been reported (Michalk et al., 1997). Prawns in the 24-h hypoxia group also had lower taurine and 2-hydroxybutanoic acid levels than the control group prawns; this was correlated with the synthesis of glutathione, a well-known antioxidant. This could explain why in previous studies, *L. vannamei* under hypoxic group showed higher antioxidant ability than that of the normoxic group (Parrilla-Taylor and Zenteno-Savín, 2011; Li et al., 2016).

Choline is the precursor of the osmolyte betaine, and is a substrate in the choline kinase-catalyzed conversion of ATP to phosphocholine and ADP. Here, the choline and betaine contents decreased in the hepatopancreas after hypoxic stress, suggesting that hypoxia may affect molecular pathways of the methionine cycle (Bertolo and McBreairty, 2013). Similar to the results of the present study, a higher flux toward the methionine cycle after environmental stress was demonstrated for gilthead sea bream *Sparus aurata* (Richard et al., 2016), the Manila clam *Ruditapes philippinarum* (Zhang et al., 2017), and rainbow smelt *Osmerus mordax* (Richards and Short, 2010). Choline and betaine are among several methyl-donors in the methionine cycle. Methionine plays an important role in protecting cells against reactive oxygen species (ROS) by neutralizing free radicals (Alirezaei et al., 2012; Wu et al., 2014). A previous study in our laboratory showed that hypoxia-induced H_2_O_2_ and ROS levels (Sun et al., 2017) and apoptosis were increased in hemocytes following hypoxia, presumably due to increased ROS production (Sun et al., 2016b). Taken together, the findings suggest that hypoxia leads to oxidative stress (Chen et al., 2017).

Thus, lower choline and betaine concentrations in *M. nipponense* in response to hypoxia may explain why *M. nipponense* is a hypoxia-sensitive prawn. Furthermore, higher pyroglutamic acid and *n*-ethylmaleamic acid levels were observed in the hepatopancreas of *M. nipponense* after hypoxia then reoxygenation for 6 h; these molecules are precursors in glutathione synthesis in the hepatopancreas of crustaceans (Niedzwiecka et al., 2011), which supports the hypothesis that antioxidant synthesis in the hepatopancreas occurs in prawns to help alleviate oxidative stress during recovery from hypoxia.

**Figure 6:**
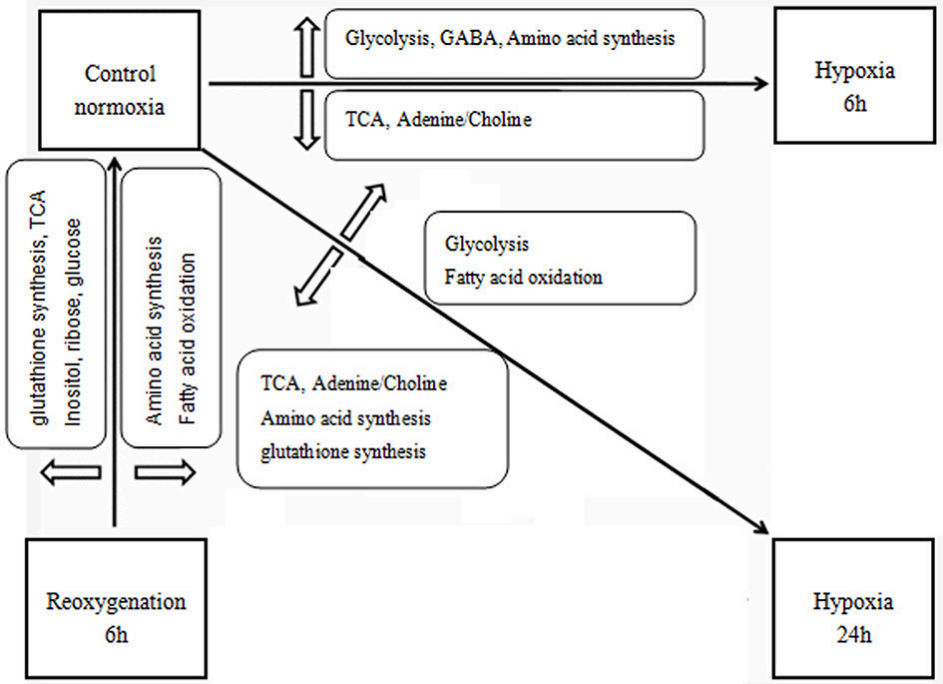
Schematic overview of the metabolic changes in the hepatopancreas of juvenile oriental river prawn in response to hypoxia (6 or 24 h) and subsequent 6-h reoxygenation. Metabolite TCA, tricarboxylic acid cycle; NAA, N-acetylaspartate, GABA, gamma-aminobutyric acid.

The significantly affected metabolites and pathways in the metabolic response of prawns to hypoxia and reoxygenation are shown in Figures 4, 6. According to the metabolic pathway analysis, a few very important pathways present distinct differences in the control vs. 6-h hypoxia groups and the control vs. 24-h hypoxia groups. For example, the valine, leucine and isoleucine biosynthesis and linoleic acid metabolism pathways are the key different metabolic pathways involved in energy supply. These results further reiterate that hypoxic prawns make use of amino acids and fatty acid metabolism to supply energy, with lower efficiency than aerobic metabolism.

In summary, hypoxia has a significant effect on antioxidant defense factors as well as energy metabolism. These findings indicate that *M. nipponense* can be a sensitive bioindicator of hypoxic stress.

## Author contributions

SS, ZBG, and HF: Conceived and designed the experiments; SS, ZBG, JZ, and ZMG: Carried out the experiments and analyzed the data; SS, HF, and XG: Supervised the project; SS: Wrote the manuscript and all authors reviewed the manuscript.

### Conflict of interest statement

The authors declare that the research was conducted in the absence of any commercial or financial relationships that could be construed as a potential conflict of interest.

## Supplementary material

Figure S1 Representative gas chromatography-mass spectrometry (GC-MS) total ion chromatograms from oriental river prawn (*M. nipponense*) hepatopancreas samples obtained from the control group (black), 6-h hypoxia treatment group (red), 24-h hypoxia group (blue), and the 24-h hypoxia followed by 6-h reoxygenation group (green).
