# Supplementary figures and images for: Based on the Metabolomic Approach the Energy Metabolism Responses of Oriental River Prawn Macrobrachium nipponense Hepatopancreas to Acute Hypoxia and Reoxygenation
Source: Front Physiol. 2018 Apr 9;9:76. doi: 10.3389/fphys.2018.00076 (PMC5900017; doi:10.3389/fphys.2018.00076)

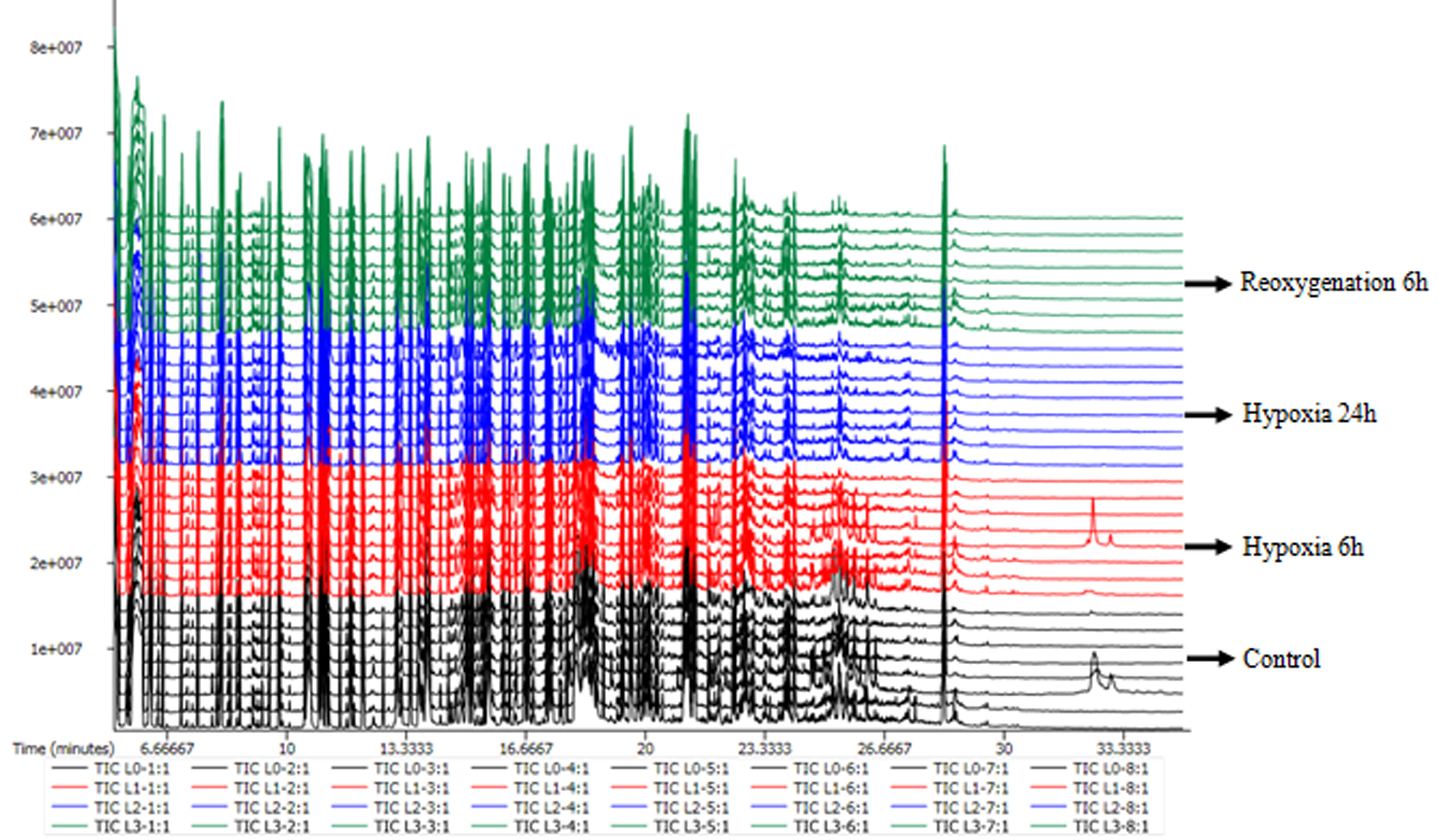

Supplement: Figure S1 — Representative gas chromatography-mass spectrometry (GC-MS) total ion chromatograms from oriental river prawn (M. nipponense) hepatopancreas samples obtained from the control group (black), 6-h hypoxia treatment group (red), 24-h hypoxia group (blue), and the 24-h hypoxia followed by 6-h reoxygenation group (green). [file Image1.TIF]
